# Examining the role of soft dimensions on the implementation of ISO 14000 environmental management systems: a graph-theoretic approach

**DOI:** 10.1007/s10479-022-04898-8

**Published:** 2022-09-06

**Authors:** Syed Mithun Ali, H. M. Belal, Sanjeeb Roy, Md. Tayabur Rahman, Ahmed Shoyeb Raihan

**Affiliations:** 1 Department of Industrial and Production Engineering, Bangladesh University of Engineering and Technology, Dhaka, 1000 Bangladesh; 2 School of Business and Management, Liverpool Business School, Faculty of Business and Law, Liverpool John Moores University, Liverpool, England

**Keywords:** ISO 14001, EMS, Human resource factor, Graph-theoretic and matrix approach (GTMA), Emerging economy

## Abstract

Organizations worldwide are now embracing different standards while approaching environmental management systems (EMS) to be environmentally and economically sustainable. The ISO 14001 EMS has captured much attention by offering efficient environmental practices organization-wide and throughout the supply chain. Human factors (HFs) are crucial behind implementing ISO 14001 EMS since research articles about ISO 14001 strongly emphasize different HFs. This study proposes a framework that solely focuses on those HFs. Influential HFs are extracted through content analysis of related literature and categorized into 5 main HFs. Opinions from experts in the relevant field about interrelationships and inheritances of the HFs are collected and converted into quantitative data. Incorporating the graph theoretic and matrix approach (GTMA), the data delivered permanent values corresponding to the main HFs and their best and worst possible values. Among the main HFs, EMS Training has been found to have the most scopes for improvement, followed by Employee Empowerment and EMS Teamwork. This study demonstrates a framework about how to assess the role of HFs behind internalizing ISO 14001 EMS and enables organizations to go for competitive benchmarking and to define and adjust goals for environmental management.

## Introduction

Globalization and industrialization have rapidly increased over the last few decades, leading to robust economic development and industrial growth (Chowdhury et al., 2018; Ndiaya & Lv, 2018). However, although the prolific growth of manufacturing firms helps improve lives, it has also caused severe environmental degradation due to the spread and accumulation of harmful wastes (Karaduman, 2022). Therefore, increasingly considerable pressure is created from the governments and NGOs that require manufacturing firms to address the environmental impacts (Ole et al., 2021). The concept of sustainability has thus emerged and has recently become the focal point for almost all organizations (Bravi et al., 2020; Colapinto et al., 2020). This concept implies a development that fulfils the needs of the present generation and considers as well as ensures the future generation’s capability of fulfilling the needs without facing difficulties (WCED, 1987). Apart from complying with the environmental regulations to preserve nature, enterprises are embracing sustainable practices to make their business processes more efficient and to get leverage from governments and customers who prefer firms with sustainable integrated systems (Abid et al., 2021; Roy et al., 2020). Consequently, the enterprises are adopting standard environmental management systems (EMS)—communication tools that are voluntary based on complying with environmental indicators to participate and minimize their negative impact (Arocena et al., 2021; Murmura et al., 2016). Especially the ISO 14001, a rigorous EMS, has been widely adopted (Lira et al., 2021; Sorooshian et al., 2018).

Scholars and practitioners have found benefits from the successful adoption of ISO 14001, such as improved environmental management practices (Boiral et al., 2018; Zimon et al., 2021), improved environmental regulatory compliance (Castillo-Martinez et al., 2021), and achieving sustainability in the supply chain (Nguyen & Hens, 2015). Furthermore, ISO 14001 helps firms improve their environmental performance (Boiral et al., 2018; Sivaprakasam et al., 2015) by improving the health and safety of the employees (Nguyen & Hens, 2015), reducing environmental risks and accidents (Djekic et al., 2014), ameliorating the waste management process (Woellner, 2020; Hasan & Chan, 2014) and reducing energy and resource consumption (Martín-peña et al., 2014). The ISO 14001 EMS also helps to improve firms' brand image and reputation (Kwon et al., 2021), maintaining solid relationships with stakeholders (Patón-Romero et al., 2019). This EMS develops employees’ competencies to get more involved in environmental activities (Jirawuttinunt & Limsuwan, 2019).

Bangladesh National Industrial Policy recommended using EMS (DoE, 2010) to eradicate the devastating impacts of pollution brought about by unplanned industrialization (Islam et al., 2018). Nevertheless, there is a significant lack of successful adoption of ISO 14001 EMS in the entrepreneurial community of Bangladesh, so they have failed to exploit the benefits of being environmentally and economically sustainable (Haque & Sharif, 2021).

Some factors influencing the successful adoption of ISO 14001 have already been discussed in the existing literature, including critical success factors (Cassells et al., 2011; Wulandari et al., 2012), motivators (Mas-Machuca & Marimon, 2019; Santos et al., 2016; Sorooshian et al., 2018; Waxin et al., 2020), challenges (Granly & Welo, 2014; Sorooshian et al., 2018; Waxin et al., 2020) and benefits (Di Noia & Nicoletti, 2016; Sorooshian et al., 2018). However, few studies have discussed the role of human factors (HFs) in successfully adopting ISO 14001 (Boiral et al., 2018; Waxin et al., 2020). Therefore, further study is needed on the mentioned topic. Likewise, past studies have also indicated the critical role of human resources (HR) factors in adopting the total quality management system (Chowdhury et al., 2018; Grover et al., 2006) and in achieving a sustainable humanitarian supply network (de Camargo Fiorini et al., 2021). Thus, it can be argued that HFs play a crucial role in successfully adopting ISO 14001 EMS (Daily & Bishop, 2003; Kivinda et al., 2021).

More specifically, according to Kaur (2011), Wee and Quazi's (2005) work is one of the first studies identifying the success factors underlying the ISO 14001 adoption. These scholars identified 7 factors, the first 3 being HFs, i.e., top management commitment, involvement of employees, and training. Besides, Daily et al. (2007) indicated the importance of HFs compared to other elements for adopting EMS and then classified HFs into 5 categories: management support, training, rewards, empowerment, and teamwork. Again, Jabbour et al. (2010) identified two HR management dimensions (i.e., functional and competitive) crucially impacting Brazil firms' EMS adoption. The positive linkage between 3 HFs, i.e., top management support, training, and rewards, and the effectiveness of EMS adoption is pointed out by Tung et al. (2014). In short, past studies showed the prominence of the HFs in influencing a successful ISO 14001 EMS adoption (Mavi et al., 2012; Sambasivan & Fei, 2008).

Although HFs have an essential role in efficient EMS implementation, studies solely integrating those factors and EMS in a complete way are incredibly scarce, especially in the Bangladesh context. Besides, the current literature shows several studies that are directly or indirectly related to HFs, e.g., Neves et al. (2017), Sujatha and Basu (2013), Kaur (2011), Oliveira & Pinheiro (2009), Sammalisto and Brorson (2008), Daily et al. (2007), Daily and Huang (2001), Delmas (2001). However, to date, to the authors’ best knowledge, no study demonstrates the hierarchical relations among various HFs and investigates their presence in organizations adopting ISO 14001. Given the critical role of HFs in implementing ISO 14001 EMS, organizing them into broad categories and analyzing their presence is vital (Rohati et al., 2017; Waxin et al., 2020). Moreover, since the necessity of an EMS is on the rise in covid-19 pandemic to boost the circular economy (Khan et al., 2021) and the increasing difficulty in maintaining ISO14001 EMS in post covid times (Ikram et al., 2022), this study is believed to play a crucial role to guide towards successful adoption of ISO14001 EMS introducing a reinforced focus on associated HFs. Therefore, the present study addresses the following research questions (RQs).

### RQ1

What are the human factors affecting the adoption of ISO 14001 EMS?

### RQ2

How the presence of these human factors be investigated quantitatively during the adoption of ISO 14001 in Bangladeshi firms?

This study identifies the vital HFs in organizations adopting ISO 14001 EMS and categorizes them into broader categories, referring to here as main HFs. The mixed-method approach is applied. The exploratory studies, with participants being HR, environment, safety and health (EHS) experts, and dedicated management employees from Bangladesh’s diverse firms, aim to explore the interdependencies among HFs and their inheritances. Afterwards, a graph theoretic and matrix approach (GTMA) is applied to examine the extent of presence, best and worst possible values of the main HFs, and the overall impact of combining all the HFs (HF Index). These kinds of multi-attribute decision-making (MADM) problems can be solved using several tools, i.e., technique for order of preference by similarity to ideal solution (TOPSIS), analytical hierarchy process (AHP), analytical network process (ANP). AHP and TOPSIS work better with independent attributes. ANP is unable to represent hierarchical relationships among attributes. On the contrary, GTMA captures hierarchical relationships of attributes or factors by considering their interdependencies and influences. Additionally, GTMA enables researchers to quantify the presence of factors and the combined effect of the factors in a management environment by exploiting experts’ opinions. (Anand & Bahinipati, 2012; Zhuang et al., 2018) However, GTMA approaches a less detailed manner in dealing with each alternative in a decision-making problem than AHP and ANP (Tuljak-Suban & Bajec, 2020). It is claimed that “the combinatorial mathematics-based approach (CMBA) is better than GTMA because of its more systematic approach to assigning relative importance in relation facilitated by consistency ratio checkup” (Rao, 2007, p. 245). Nevertheless, GTMA avoids complicated and lengthy numerical manipulations and these traits are emphasized more in trade-off accepting the probability of some minor deviation in the result in this study.

Further discussions of this study are structured in the following way. Highlighted works of literature on ISO 14001 EMS, the identified HFs, reviewed relevant to this study, and existing research gaps and contributions have been discussed in Sect. 2. Section 3 involved describing theoretical backgrounds, the proposed framework and the application of the framework. Obtained quantitative information after applying the framework is analyzed, and how this study adds value to the research streams is elaborated in Sect. 4. Finally, this study's limitations and future scopes are communicated in Sect. 5.

## Literature review

ISO 14001 was firstly introduced over twenty years ago (Sartor et al., 2019). Many researchers have worked on ISO 14001 from diversified perspectives. In response to the current trend to form integrated management system, several studies discussed the mechanism that integrates ISO 14001 with ISO 9001, OHSAS 18,001, ISO 22000, ISO 32000, ISO 45001 (Alfredo & Nurcahyo, 2018; Muzaimi et al., 2017; Purwanto et al., 2020). Again, many scholars worked on the assessment of environmental performance due to ISO 14001 adoption (Aravind & Christmann, 2011; Arocena et al., 2021; Bahmed et al., 2009; Põder, 2006; Seiffert, 2008; Sivaprakasam et al., 2015) and indicators of the effectiveness of ISO 14001 adoption (Abid et al., 2021; Campos et al., 2015; Rino & Salvador, 2017). Some studies examined the relationships of ISO 14001 with ISO 9001 (Santos et al., 2016; Tarí et al., 2012), information system (Fiorini et al., 2019), operations (González-Benito & González-Benito, 2008; Treacy et al., 2019), lean manufacturing (Habidin et al., 2018). Besides, how ISO 14001 is internalized in small and medium enterprises (SMEs) (Nee, 2011) and universities (Halila & Tell, 2013) or the contrast of environmental performance of ISO 14001 certified companies (Yin & Schmeidler, 2009) are contemplated. Again, multiple studies attempted to explore the significance of different HFs in implementing ISO 14001 (Sujatha & Basu, 2013; Waxin et al., 2020). Moreover, how differences in culture in diverse countries can impact the adoption process of EMS has been discussed in recent studies. One of these studies, using hypothesis testing, has found that cultural positivity accelerates the EMS implementation process. At the same time, the maturity of EMS implementation decouples this leverage of national culture (Orcos & Palomas, 2019). Another study discussed how cultural differences among nations indirectly affect creating a different level of environmental performance because there exist correlations between the 3 environmental management practices: internal review, sourcing review and environmental management system with environmental performance (Song et al., 2018).

Some articles examined the role of HFs on environmental performance. Daily et al. (2007), Babakri et al. (2003), and Kitazawa and Sarkis (2000) analyzed the correlation between HFs and environmental performance, identified training and awareness programs as essential factors and examined the role of power delegation, commitment, participation to implement ISO 14001 respectively. Further, Neves et al. (2017) concluded environmental and socio-cultural factors are crucial for the USA and Brazilian companies. For universities, commitment, organizational structure, team synergy, and guidance were suggested among the 5 major factors by Price (2005). However, interactive training was prioritized by Sammalisto and Brorson (2008). Balzarova et al. (2006) detected people, processes, and organizational structures among 4 major factors for UK factories. Zeng et al. (2005) found top management commitment, dynamic middle management, well-defined responsibilities, and policies among the top 5 critical factors for Chinese companies. Again, the role of top management commitment was analyzed by Waxin et al. (2020), Chowdhury et al. (2018) and Ejdys et al. (2016) for UAE organizations and Australian-Kiwis firms on a general note respectively. Furthermore, Rohati et al. (2017), Nee (2011), Kaur (2011) and Haslinda and Chan (2010) analyzed the significance of training, top management, review and monitoring, and empowerment in Malaysian enterprises. The commitment of top management, policies, and training was identified as the most critical factors for the Iranian cement industry by Hessami and Soleimani-Nezhad (2012). However, Oliveira & Pinheiro (2009) found communication to be another effective strategy for Brazilian companies. Again, the impact of the training was analyzed for Brazilian companies by Jabbour (2015). Sujatha and Basu (2013) noticed teamwork and training-education as the essential HFs and rewards not significantly correlated for Indian fertilizer firms. Interestingly, through a literature review, Daily and Huang (2001) also identified top management support, training, delegation of power, group work and incentive systems as the key factors.

Studying the relevant literature, multiple shortcomings in the current research streams focusing on the role of HFs in ISO 14001 have been detected. Few works of literature (Sujatha & Basu, 2013; Kaur, 2011; Oliveira & Pinheiro, 2009; Sammalisto & Brorson, 2008; Daily et al., 2007; Daily & Huang, 2001) have solely worked with HFs. One article (Neves et al., 2017) has implicitly discussed HFs, but not being comprehensive and incisive enough. Those studies have used research tools, i.e., hypothesis testing (Daily et al., 2007; Nee, 2011), SEM (Chowdhury et al., 2018; Jabbour, 2015; Rohati et al., 2017), MICMAC (Ejdys et al., 2016), TOPSIS (Hessami et al., 2012), regression analysis (Kaur, 2011; Neves et al., 2017), and descriptive research methods (Babakri et al., 2003; Balzarova et al., 2006; Daily & Huang, 2001; Kitazawa & Sarkis, 2000; Oliveira & Serra Pinheiro, 2009; Price, 2005; Sammalisto & Brorson, 2008; Sujatha & Basu, 2013; Waxin et al., 2020; Zeng et al., 2005) but failed to demonstrate the hierarchical relations among the HFs and role of each main HF category quantitatively in the successful adaptation of EMS in organizations. Some research works (Orcos & Palomas, 2019; Song et al., 2018) on environmental management practices highlighted that the national culture influences the implementation of EMS. Their focuses were limited to those cultural dimensions without having any hierarchical relationship. Additionally, since those cultural dimensions are considered to have a significant impact on environmental performance during the initiation of EMS whereas human factors seem relevant throughout all the phases EMS implementation, the study about the influence of human factors on EMS implementation holds its unique edge. One (Daily et al., 2007) among these literature has considered the interrelationship among those HFs. The above discussion reveals that no previous work examined the presence of HFs in implementing ISO EMS 14,001, considering those HFs’ hierarchical relationships, particularly in the context of an emerging economy like Bangladesh. This study has linked the gaps by contributing to the nexus of human factors and ISO 14001 literature.

## Methods

To investigate the presence of the HFs behind implementing the ISO 14001 EMS, GTMA has been adopted. GTMA is a powerful approach to modelling and analyzing a system of multiple attributes (Agrawal et al., 2016; Gupta & Singh, 2015). Consequently, this tool has been applied in versatile research problems: assessing critical drivers of Lean Six Sigma (Lande et al., 2019), analyzing supply chain complexity quantitively (Kavilal et al., 2018), evaluating alternatives for reverse logistics (Agrawal et al., 2016) to name but a few.

GTMA consists of 3 basic steps- digraph representation for visual analysis of the interdependencies of the factors; matrix representation consisting of those interdependencies among factors and inheritances (influence) of individual factors. And finally, the permanent representation depicts the significance of all the factors by a single numerical index that enables one to compare, rank and select the optimum factors (Agrawal et al., 2016).

**Fig. 1:**
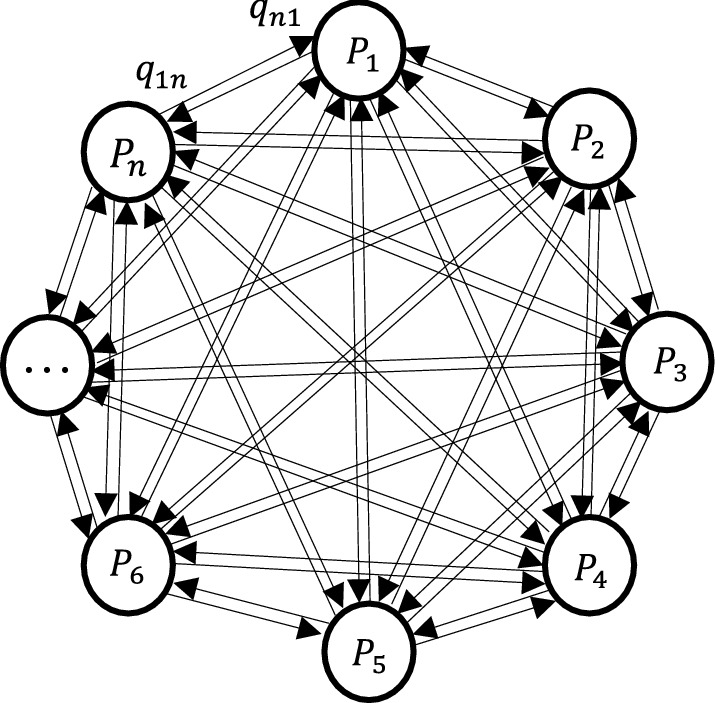
Diagraph for ‘n’ number of factors in a system

Diagraph consists of nodes and directed edges or arcs connecting the nodes. An example is illustrated in Fig. 1, where each of the nodes ${P}_{1}$, ${P}_{2}$, ${P}_{3}$, …, ${P}_{n}$ represents a factor or attribute influencing any decision-making scenario. The arcs indicate the interdependencies among the nodes in pairwise form. For example, ${q}_{kl}$ indicates how factor $k$ influences factor $l$ or the extent of dependence of factor $l$ on factor $k$ represented by an arc pointing from node $k$ towards node $l$. (Agrawal et al., 2016; Grover et al., 2006).

Afterwards, matrix representation of the diagraph known as the variable permanent matrix (VPM) is constructed to depict all interdependencies and inheritances of those factors. The VPM for the decision-making scenario of Fig. 1 has been shown in Eq. (1) (Anand & Bahinipati, 2012).

1 $$VPM=\left[\begin{array}{ccc}{P}_{1}& \dots & {q}_{1n}\\ \vdots & \ddots & \vdots \\ {q}_{n1}& \dots & {P}_{n}\end{array}\right]$$

In the VPM of Eq. (1), the diagonal entries ${P}_{1}$, …, ${P}_{n}$, indicate inheritances of all corresponding nodes and the off-diagonal entries of the matrix, ${q}_{kl}$, indicate the interdependencies (Anand & Bahinipati, 2012; Grover et al., 2006).

Finally, to generate the permanent function of the VPM, its determinant has been expanded (similar to expanding the determinant of a matrix except taking a positive sign always) in Eq. (2) (Anand & Bahinipati, 2012).

2 $$Per \left(VPM\right)=\sum_{l}\prod_{k}{P}_{i}{q}_{kl}$$

This permanent representation is the key in the pursuit of investigating the role of the HFs behind internalizing ISO 14001.

### Design of Solution Methodology

The solution methodology has been designed as follows to operationalize ISO 14000 EMS.

Step (1): Reviewing research articles from reputed scholarly sources and databases.

Step (2): Screening out most relevant HFs.

Step (3): Finalizing HFs influential for implementing ISO EMS 14,001.

Step (4): Developing a digraph combining the main HFs (main system) and diagraphs for each of those main HFs (sub-systems).

Step (5): Preparing a comprehensive and concise set of questionnaires to intake the opinion of the experts about the inheritances of individual HFs and the interdependencies of the HFs.

Step (6): Gathering all the responses from the experts and quantifying them following standard qualitative to the quantitative conversion system.

Step (7): Constructing VPMs for all the sub-systems.

Step (8): Evaluating permanent values from each of those VPMs.

Step (9): Constructing VPM for the main system.

Step (10): Evaluating the HF Index.

Step (11): Evaluating the best-case and the worst-case permanent values of those sub-systems and ranking main HF according to their improved opportunity.

**Fig. 2:**
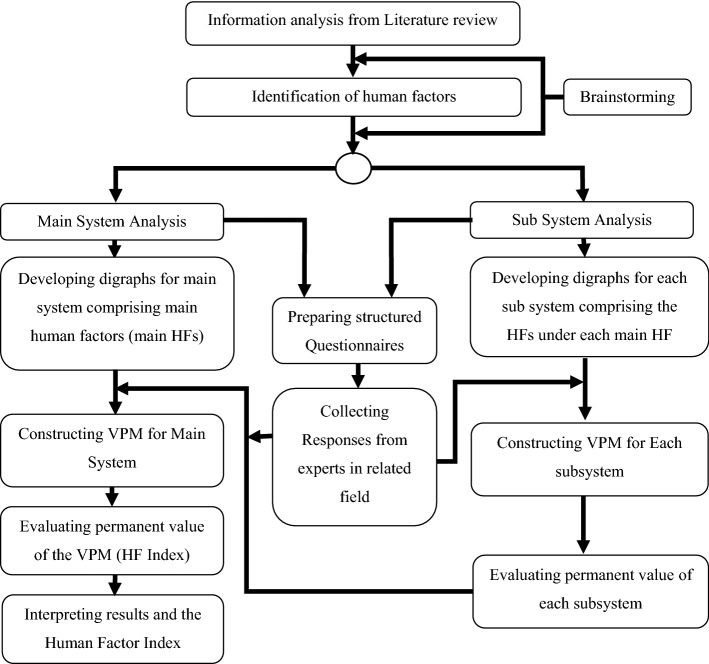
Flow chart of proposed solution methodology

The proposed solution methodology is shown in Fig. 2.

### Application of proposed solution methodology

This study used the context of Bangladesh to examine the role of HFS in implementing ISO 14001 EMS. First, we identified and finalized the factors for implementing ISO 14001 EMS (steps 1–3 mentioned above). Surfing ScienceDirect, Web of Science (WoS), Scopus and Google Scholar databases with keywords, i.e., ‘human/cultural $+$ factors/behaviors/aspects $\pm$ ISO 14001 $\pm$ EMS’ or simply ‘ISO 14001’, 150+ articles were accumulated from reputed journals and proceedings. Subsequently, a thorough content analysis was performed based on criteria, i.e., (1) written in English, (2) peer-reviewed, (3) relating HFs with ISO 14001, to sort the most relevant studies. This sorting narrowed down 24 papers that were found to be instrumental in corporealizing all the 19 HFs of this study by going through multiple brainstorming sessions. Additionally, 4 HFs were proposed after those sessions to ensure the existence of all the crucial HFs. Furthermore, those HFs were required to be categorized due to having many common grounds with each other. Fortunately, among the papers on the shortlist, Sujatha and Basu (2013), Kaur (2011), Oliveira & Pinheiro (2009) were found to introduce different categorization approaches. However, the most comprehensive categorization of an influential research work by Daily et al. (2007) about HFs’ impact on EMS is adopted here. Thus, those 23 HFs are categorized into 5 main HFs i.e., top management involvement, EMS teamwork, employee empowerment, EMS training, EMS rewards. All these HFs with their corresponding short descriptions and sources are enlisted in Table 1.

**Table 1:** Human factors with their assigned notation, description, and sources

Main Human Factor	Human Factor	Notation	Description	Sources
Top management Involvement (${F}^{1}$)	Commitment and Support	${F}_{1}^{1}$	Top management’s commitment and support ensure proper resource allocation and an encouraging environment	Chowdhury et al. (2018), Ejdys et al. (2016)
	Management review	${F}_{2}^{1}$	Recurrent reviews to assess the endeavors helps in adjusting the discrepancies and boosting the overall process	Hörisch et al. (2020), dos Santos Ferreira et al. (2019)
	Environmental policy and objectives	${F}_{3}^{1}$	Setting effective policies and objectives creates common value, generating performance consistency	Wang and Zhao (2020)
EMS Teamwork (${F}^{2}$)	Mutual support	${F}_{1}^{2}$	Supporting each other in the team helps to build up synergic progression	Mwita & Mwakasangula (2020); Sorooshian and Ting (2018)
	Effort	${F}_{2}^{2}$	Efforts from teammates accelerate progress	Chen et al. (2020), Junsheng et al. (2020)
	Cohesion	${F}_{3}^{2}$	Building a bond among teammates helps them stick together to achieve goals	Proposed
	Co-ordination	${F}_{4}^{2}$	Co-ordination of individual efforts ensures consistency and reduces completion time	Sorooshian and Ting (2018)
	Communication	${F}_{5}^{2}$	Communication among teammates assists in resolving conflicts, innovating ideas and correcting misguided procedures	Fonseca and Domingues (2018)
Employee Empowerment (${F}^{3}$)	Problem-solving skills	${F}_{1}^{3}$	This enables the personnel to solve problems on a root level	Mwita & Mwakasangula (2020), Madani et al. (2017), Virapongse et al. (2016)
	Self-control	${F}_{2}^{3}$	Self-control ensures the enactment of an individual’s power rightfully	Proposed
	Individual Thinking	${F}_{3}^{3}$	Enhancement of thinking ability using own knowledge and perception assists in generating innovative approaches	Junsheng et al. (2020)
	Self-reliance	${F}_{4}^{3}$	Relying on own ensures proper distribution of workloads and the creation of specialists for different tasks	Sorooshian et al. (2018)
EMS Training (${F}^{4}$)	Attitude Change	${F}_{1}^{4}$	Acknowledging duties as crucial and productive, personnel can engage their focus effectively	Chen et al. (2020)
	Awareness	${F}_{2}^{4}$	Awareness about the prescribed way to conduct activities and the consequences of not following them would ensure the workflow is consistent	Bravi et al. (2020)
	Proactive attitude	${F}_{3}^{4}$	Sensing the possible sources of non-conformities and allocating reinforcements would reduce the time and resources required	Mungai et al. (2020), Peiró-Signes et al. (2020)
	Willingness to participate	${F}_{4}^{4}$	Voluntary engagement of personnel will accelerate their learning progress and eventually result in cross-functional teams	Adriana et al. (2020), Hameed et al. (2020)
	Non-conformity Reporting	${F}_{5}^{4}$	Monitoring and giving feedback will help readjust the process briefly and reduce the waste of resources	Budi et al. (2020), Voukkali et al. (2017)
	Education	${F}_{6}^{4}$	Education about sustainability and technology management would enable personnel to visualize the big picture	Zhuravskaya et al. (2016)
	Adaptability	${F}_{7}^{4}$	Efficacy toward adjusting EMS tasks to conform with upcoming standards and incorporating emerging techniques would ensure continuous improvements	Proposed
EMS Rewards (${F}^{5}$)	Commitment from employees	${F}_{1}^{5}$	The engagement of personnel in EMS activities will increase the possibility of successful enforcement	Chen et al. (2020)
	Awareness about environmental policies among the employees	${F}_{2}^{5}$	Awareness about the organizational policies towards the environment would help personnel realize the necessity of accomplishing each activity	Mittal et al. (2020), Chen et al. (2020)
	Reinforcement of responsibilities	${F}_{3}^{5}$	Deployment of well-defined job duties provides scopes for innovation within the daily role	Proposed
	Motivation	${F}_{4}^{5}$	Motivation ensures the retainment of focus	Yu et al. (2020)

Among those 23 HFs in Tables 1, 4 HFs were proposed by authors and cross-verified by industry experts and academicians to capture the complete mechanism behind EMS implementation. Cohesion (1st proposed HF) creates a natural drive to connect physically and emotionally with the thoughts and activities of each team member while doing an EMS task assigned to the team, and the authors believe it to be an essential HF alongside the HFs (mutual support, effort, co-ordination and communication under EMS Teamwork). Self-control (2nd proposed HF) reflects a perfect blend of authority and sensibility in exercising power which is considered to have unique importance in employee empowerment alongside problem-solving skills, individual skills, and self-reliance. Adaptability (3rd proposed HF) generates openness to new editions in standard activities to improve EMS implementation process continually, and thus, it has worth being a separate HF under EMS Training. To prevent the situation when some employees try to outperform to achieve rewards by interfering with EMS tasks assigned to other employees from happening, reinforcement of responsibilities (last proposed HF) ensures proper boundary of each employee’s responsibility. Thus, this HF has unique significance to be included as a separate HF among other HFs (commitment, awareness, motivation) under EMS rewards.

**Fig. 3:**
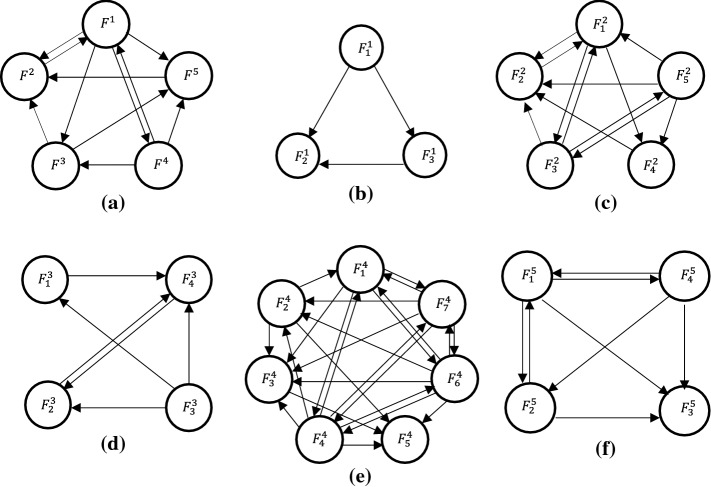
Digraphs for, main human factor (**a**), human factors- (**b**–**f**)

Step (4): At this point, interdependencies of HFs are taken under consideration. After going through multiple brainstorming sessions, some of the interdependencies are considered to be negligible, thus avoided from further consideration. This sorting of negligible interdependencies has been incorporated since they are deemed to have a meager impact on the overall result, and this ensures the length of questionnaires stays within the limit to enhance the experts’ active engagement. Afterwards, diagraphs for the main and sub-systems are constructed. The digraphs representing the main system and sub-systems are given in Fig. 3.

Step (5): Finishing diagraph construction enables to proceed for constructing VPMs of the main HFs. However, all the inheritances and interdependencies of HFs are required priorly. To evaluate them, two questionnaires were prepared.

Step (6): Experienced professionals from either HR or EHS functions and professionals ranging from executives to senior management were asked for their responses to the questionnaires respectively. Consequently, senior experts with significant experience in HR/EHS function and management professionals from ISO 14001 certified companies from diverse industries have responded to the questionnaires. Profiles of all the respondents are given in Tables 2 and 3.

**Table 2:** Profile of respondents to first questionnaire

Role	Company type	Belonging industry	Experience (in Years)
EHS	MNC	Fast moving consumer goods (FMCG)	4
EHS	MNC	FMCG	2
EHS	Local	Automobile	2
HR	MNC	FMCG	20
HR	Local	LPG gas	4
HR	MNC	Textile	17
HR	MNC and Local	Pharmaceuticals (2) and Non-profit Organizations (3)	27

**Table 3:** Profile of respondents to second questionnaire

Belonging industry	Belonging company type	Offerings type	Market size	Experience (in years)
Bank	Multinational	services	Large	2
Ceramics	Local	products	Medium	2
Sports wares	Multinational	Products	Large	3
Super shop chain	Local	Services	Large	1
Infrastructure development	Local	Services	Medium	5
Cement	Multinational	Products	Large	5
Textiles	Multinational	Products	Large	2
Renewable energy technologies	Local	Products	Medium	2
Steel	Local	Products	Large	1
Textiles	Multinational	Products	Medium	2
FMCG	Multinational	Products	Medium	4
Garments	Multinational	Products	Large	4
FMCG	Multinational	Products	Medium	6
Automobiles	Local	Products	Medium	4
FMCG	Multinational	Products	Large	2
Agrochemical	Multinational	Products	Large	12
Pharmaceuticals	Local	Products	Large	14
Garments	Multinational	Products	Large	11
Sports wares	Multinational	Products	Large	4
Apparels	Multinational	Products	Large	4
Telecommunication	Multinational	Services	Large	2
Electrical and electronic appliances	Local	Products	Large	3
Retail chain	Multinational	Products	Large	12
Agrochemical	Multinational	Products	Medium	8
Garments	Multinational	Products	Large	2

In both questionnaires, the questions were constructed concisely but comprehensively, and the options were given in linguistic variables to make it easy for the respondents to respond with spontaneity and mindfulness. These qualitative responses are converted into numerical values following the scales in Tables 4 and 5. These scales have also been followed in some influential GTMA research works (Anand & Bahinipati, 2012; Gupta & Singh, 2015; Gurumurthy et al., 2013; Kumar & Kumar, 2016; Lande et al., 2019).

**Table 4:** Scale for converting linguistic response to interdependencies

Linguistic judgement	Corresponding numerical value (${q}_{kl}$)
Very Strong	5
Strong	4
Medium	3
Weak	2
Very Weak	1

**Table 5:** Scale for converting linguistic response to inheritances

Linguistic judgement	Corresponding numerical value (${P}_{i}$)
Exceptionally low	1
Very low	2
Low	3
Below average	4
Average	5
Above average	6
High	7
Very high	8
Exceptionally high	9

After converting into numerical values, internal consistencies of the questionnaire responses are evaluated by finding values of Cronbach’s alpha using IBM’s SPSS software. These are found to be 0.964 and 0.967 respectively, both exceeding 0.7, and thus they validate the responses to the questionnaires (Butts & Michels, 2006; Olatunji et al., 2017).

Step (7): The average values of the converted numerical values from responses to all questions of the first and the second questionnaire would provide the interdependencies (off-diagonal entries represented as ${q}_{kl}$) and inheritances (diagonal entries represented as ${P}_{i}$) respectively. The following demonstrates how these numerical values are used to build all the VPMs gradually.

Corresponding average values from questions about interdependencies regarding the HFs [commitment and support (${F}_{1}^{1}$), management review (${F}_{2}^{1}$), environmental policy and objectives (${F}_{3}^{1}$)] under Top management Involvement (${F}^{1}$) are placed on the respective non-diagonal positions in the VPM of Eq. (3), e.g., placing the interdependency between ${F}_{1}^{1}$ and ${F}_{2}^{1}$ (4.14) is placed on row1-colume2 position.

3 $${F}^{1{^{\prime}}}=\left[\begin{array}{ccc}-& 4.14& 4.29\\ 0& -& 0\\ 0& 3.57& -\end{array}\right]$$

Afterwards, average values from questions about inheritances of those 3 HFs under Top management Involvement (${F}^{1}$) are placed on the respective diagonal positions in Eq. (3), e.g., placing the inheritance of ${F}_{1}^{1}$ (6.14) on row1-colume1 position. Finally, the complete VPM for the first sub-system ${F}^{1}$ is given in Eq. (4).

4 $${F}^{1}=\left[\begin{array}{c@{\quad}c@{\quad}c}6.52& 4.14& 4.29\\ 0& 6.60& 0\\ 0& 3.57& 6.64\end{array}\right]$$

Replicating the mentioned procedures, all the VPMs for the other sub-systems (${F}^{2}, {F}^{3}, {F}^{4}, {F}^{5}$) are derived and given in Eqs. (5), (6), (7), (8):

5 $${F}^{2}=\left[\begin{array}{ccc}\begin{array}{cc}6.32&\quad 3.71\\ 3.71&\quad 6.80\end{array}& \begin{array}{cc}4&\quad\qquad  4\\ 0&\qquad \quad 0\end{array}& \begin{array}{c}0\\ 0\end{array}\\ \begin{array}{cc} 4&\quad\qquad 3.57\\ 0&\quad\qquad  3.86\end{array}&\quad  \begin{array}{cc}6.04& 0\\ 0&\quad 6.32\end{array}&\quad \begin{array}{c}3.57\\ 0\end{array}\\ \begin{array}{cc}4.43&\quad 3.86\end{array}& \begin{array}{cc}4.00&\quad 4.29\end{array}&\quad 6.72\end{array}\right]$$

6 $${F}^{3}=\left[\begin{array}{c@{\quad}c}\begin{array}{c@{\quad}c}6.44& 0\\ 0& 6.28\end{array}& \begin{array}{cc} 0& 3.43\\ 0& 3\end{array}\\ \begin{array}{cc}3.43& 3.14\\ 0& 0\end{array}& \begin{array}{cc}5.96& 3\\ 3.29& 6.12\end{array}\end{array}\right]$$

7 $$\left[ \begin{array} {ccccccc} 6.44  & 0 & 3.43 & 3.57 & 3.57 & 3.14 &3.14 \\ 3.14 & 6.56 & 3.43 & 0 &3.14 & 0 & 0 \\ 0 & 0& 6.32 & 0 & 3.57 & 0 & 0\\  3.29 & 3.86 &3.29 &6.56 & 3.43 & 3 & 3.29 \\ 0 & 0 & 0 & 0 & 6.32 & 0 & 0 \\ 3.29 & 3.57 & 3.43 & 3.14 & 3.57 & 6.56 & 3.29 \\ 3.43 & 3.57 & 3 & 3 & 0&0& 6.16 \end{array} \right]$$

8 $${F}^{5}=\left[\begin{array}{c@{\quad}c}\begin{array}{c@{\quad}c}7.08& 3.43\\ 3.14& 6.68\end{array}& \begin{array}{cc}3.43& 3.29\\ 3.43& 0\end{array}\\ \begin{array}{cc}0& 0\\ 3.29& 3.29\end{array}& \begin{array}{cc}6.40& 0\\ 3.14& 6.60\end{array}\end{array}\right]$$

Due to the inability to find the main HFs' inheritances until this step, the incomplete VPM (with the interdependencies found in this step) for the main system is given in Eq. (9).

9 $$F=\left[ \begin{array} {cccccc} -  & 4.43 & 4.43 & 4.43 &4.29 \\ 3.57 & - & 0 & 0 & 0 & \\ 0 & 3.43& - & 0 & 3 & \\  3.71 & 3.43 &3.57 &- & 3.14 &  \\ 0 & 3.43 & 0 & 0 & -  \end{array} \right]$$

Those inheritances in Eq. (9) will be filled with permanent values of the VPMs of the sub-systems mentioned in Eqs. (4), 5, 6, 7, (8).

Step (8): To evaluate the permanent value of any VPM with any order, a code in C language using Code::Blocks has been generated following the general expression of Eq. (2) and using the permanent values of the VPMs of all the sub-systems [given in Eq. (4) to Eq. (8)] are evaluated and mentioned in Eq. (10), (11), (12), (13, )(14).

10 $$Per\left({F}^{1}\right)= 285.73$$

11 $$Per\left({F}^{2}\right)=34578.04$$

12 $$Per\left({F}^{3}\right)=2317.01$$

13 $$Per\left({F}^{4}\right)=1943433.88$$

14 $$Per\left({F}^{5}\right)=3132.92$$

Step (9): Those permanent values of Eq. (10), (11), (12), (13), (14) are put into diagonal positions respectively in Eq. (9), and thus, the complete VPM for the main system is found, which is given in Eq. (15).

15 $$F=\left[\begin{array}{ccccc}285.73& 4.43 & 4.43 & 4.43 & 4.29 \\ 3.57& 34578.04 & 0 & 0 &0 \\ 0 & 3.43 & 2317.01 & 0 & 3 \\ 3.71 & 3.43 & 3.57 & 1943422.88 & 3.14 \\ 0 & 3.43 & 0 & 0 & 3132.92 \end{array}\right]$$

Step (10): Using the same code used in Step (8), the permanent value of the VPM of Eq. (15) representing the main system is evaluated and given in Eq. (16).

16 $$HF Index=Per\left(F\right)=1.39 \times {10}^{20}$$

Therefore, $1.39 \times {10}^{20}$ is the overall HF index in the context of the diverse industries of Bangladesh that attempt to internalize ISO 14001.

Step (11): At this point, HF index corresponding to the best and worst possible situation can be found by assuming the best and worst possible values of inheritances of the main system respectively. Since the inheritances of the main system are derived from the permanents of the sub-system VPMs, therefore, when the inheritances of the sub-systems are assumed to be maximum (minimum) [taking management is giving maximum (least) importance to all the HFs respectively], the permanents of the corresponding sub-systems’ VPMs are the best-possible (worst-possible) inheritances of the main system (Grover et al., 2006). For example, the best possible value of the first inheritance of the main system can be found considering the best-possible inheritances in the VPM of the first sub-system in Eq. (4). The resulting VPM and its permanent value are given in Eqs. (17) and (18) respectively.

17 $${F}^{1Pmax}=\left[\begin{array}{ccc}9& 4.14& 4.29\\ 0& 9& 0\\ 0& 3.57& 9\end{array}\right]$$

18 $$Per({F}^{1Pmax})=729$$

Similarly, the best-possible values of other inheritances of the main system are found, and the values are given in Table 4. Those are fed as the diagonal entries in Eq. (9). The resulting VPM and its permanent value (best-possible HF Index) are given in Eqs. (19) and (20) respectively.

19 $${F}^{pmax}=\left[\begin{array}{ccccc}729 & 4.43 & 4.43  & 4.43 & 4.29 \\ 3.57 & 110517.14 & 0 & 0 &0 \\ 0 & 3.43 & 7987.76 & 0 & 3 \\ 3.71 & 3.43 & 3.57 & 11016168 & 3.14 \\ 0 & 3.43 & 0 & 0 & 8616.03 \end{array}\right]$$

20 $${HFI}^{pmax}=Per\left({F}^{pmax}\right)=6.11 \times {10}^{22}$$

Likewise, the worst-possible value of the first inheritance of the main system can be found by considering the minimum inheritances in the VPM of the first sub-system in Eq. (4). The resulting VPM and its permanent value are given in Eqs. (21) and (22) respectively.

21 $${F}^{1Pmin}=\left[\begin{array}{ccc}1& 4.14& 4.29\\ 0& 1& 0\\ 0& 3.57& 1\end{array}\right]$$

22 $$Per({F}^{1Pmin})=1$$

Also, the worst-possible values of the other inheritances of the main system are calculated and given in Table 6. Those values are fed as the diagonal entries in Eq. (9). The resulting VPM and its permanent value (worst-possible HF Index) are given in Eqs. (23) and (24), respectively.

**Table 6:** Permanent values for sub-systems/system and Improvement opportunity

Subsystem/ system	Permanent value for the current situation	Permanent best-possible situation	Permanent worst-possible situation	Existing improvement opportunity in percentage
$({F}^{1})$	285.73	729	1	60.88
$({F}^{2})$	34,578.04	110,517.14	2314.9	70.18
$({F}^{3})$	2317.01	7987.76	80.57	71.72
$({F}^{4})$	1,943,422.88	11,016,168	3732.5	82.39
$({F}^{5})$	3132.93	8616.03	56.58	64.06
$HF Index$ or $F$	$1.39\times {10}^{20}$	$6.11\times {10}^{22}$	$3.99\times {10}^{10}$	99.80

23 $${F}^{pmin}=\left[\begin{array}{ccccc} 1 & 4.43 & 4.43 & 4.43 & 4.29 \\ 3.57 & 2314.90 & 0 & 0 & 0 \\ 0 & 3.43 & 80.57 & 0 & 3 \\ 3.71 & 3.43 & 3.57 & 3732.50 & 3.14 \\ 0 & 3.43 & 0 & 0 & 56.58 \end{array}\right]$$

24 $${HFI}^{pmin}=Per\left({F}^{pmin}\right)=3.99\times {10}^{10}$$

## Results and discussions

The permanent values in Table 6 play a pivotal role in discovering significant interpretations about the HFs regarding their role in internalizing ISO 14001.

Firstly, those permanent values give insights into an organization intended to implement ISO 14001 EMS in a quantitative manner that helps to identify relative position by comparing their permanent values and HF Indices with other companies from similar industries in a particular time. Secondly, using the permanent values for an observed, best-possible and worst-possible situation, ‘improvement opportunity’ for each of the main HFs can be evaluated using Eq. (25) that has been adopted from a previous influential research work by Kumar and Kumar (2016).

25 $${Improvement\,\, Opportunity }_{of\,\, attribute}=\frac{best\,\, possible\,\, value-observed\,\, value }{best\,\, possible\,\, value-worst\,\, possible\,\, value}$$

The Equation is used here to assess the improvement opportunity for all the main HFs, which are given in Table 6. Among the main HFs, EMS Training has the most improvement opportunity (82.39%) for internalizing ISO 14001 at that observed time frame followed by other main HFs: Employee Empowerment (71.72%), EMS Teamwork (70.18%), EMS Rewards (64.06%), Top Management Involvement (60.88%). Those improvement opportunity values help keep track of improvement or lag by comparing those values from different time frames while having internal or external auditing.

According to the values of improvement opportunity in Table 6, EMS training appears to be this study's most critical main HF. Interestingly, the works of Rohati et al. (2017), Jabbour (2015), Sammalisto and Brorson (2008), Babakri et al. (2003) based on their case studies regarding manufacturing companies from Malaysia, Brazilian companies, a Swedish university and industrial companies from the U.S. respectively did prioritize training than any other HFs for ISO 14001 implementation. However, Hessami and Soleimani-Nezhad (2012), Kaur (2011) and Zeng et al. (2005) highlighted top management commitment in the first place among all the HFs in their work regarding the Iranian cement industry. On the contrary, Malaysian corporations and Chinese companies are opposite to the findings of this study since Top Management Involvement carries the least value of improvement opportunity. Again, Employee Empowerment, the second most crucial main HF that this study found, was mentioned to play the lead role among all HFs by Kaur (2011) based on Malaysian companies. Although EMS teamwork has been found out to be the third significant main HFs, studies i.e., Sujatha and Basu (2013) and Daily et al. (2007) founded teamwork more influential than other HFs based on their case studies in fertilizer farms of India and U.S. aerospace sector respectively. Interestingly, EMS reward, that has been founded to be the second least significant main HFs, was also regarded less significant than other HFs in the work of Kaur (2011). Therefore, it can be realized that varying industry, geographical boundary, and even time frame results in different findings regarding the answer of the question “which HF is the most crucial one to put greater efforts”. For Bangladeshi business organizations, this study concludes with EMS training to be the most potential HF that need more attention; Employee Empowerment and EMS Teamwork to be addressed with moderate focus; and EMS Rewards and Top Management Involvement to have already sufficient resources and concentration and thus requiring least attention.

Thus, the emergence of the permanent values and improvement opportunities in this study turn the intangible problem of addressing HFs for internalizing ISO 14001 into a quantitative problem. Finally, this study summarizes the following implications to benefit practitioners and researchers.

Industry professionals can adopt this study to establish guidelines to assess the role of HFs in their organizations with the help of internal employees and to accomplish competitive benchmarking through quantitative analysis of survey data regarding renowned companies from the same industry for setting goals to reach the level. And this is how the organization enables the successful internalization of ISO 14001. This study, in its process, sorts the HFs that have more potential for improvement, helping managers prioritize their focus and resources. Researchers can follow the framework to work with HFs of EMS in a generic manner considering a broad spectrum of industries within other geographic boundaries. Furthermore, this analysis could be replicated for other standard systems, e.g., OHSAS 18,001, ISO 45001 and certification processes, e.g., LEED, Higg, in future.

## Conclusions

This study is the first of its kind to identify HFs crucial behind implementing ISO 14001 EMS, investigate the quantitative information of those HFs, and pinpoint the HFs requiring more attention by exploiting the GTMA research tool. At first, 5 main HFs and, under them, 19 HFs were identified from 21 influential research articles after reviewing relevant literature extracted from scholarly sources and subsequently, 4 HFs were proposed after going through multiple intensive brainstorming sessions. Afterwards, two questionnaires were prepared from those HFs, responses from 7 industry experts (ranging in experience from 2 to 27) and professionals (from 16 different industries with expertise ranging from 2 to 14 years) from Bangladesh were collected and transformed into quantitative information. Likewise, the quantitative data from questionnaires was incorporated in GTMA to get quantitative insights regarding the presence and improvement opportunity potential of those 5 HFs in internalizing ISO14001 EMS in the business organization around Bangladesh. Finally, the generated results from this study are attempted to be communicated by exposing the contrasts and similarities with results from relevant studies (Daily et al., 2007; Hessami & Soleimani-Nezhad, 2012; Kaur, 2011; Sujatha & Basu, 2013; Zeng et al., 2005) and practical and research implications of this study.

The previous studies assessing different HFs for successful adoption of EMS have used different MCDM tools, i.e., hypothesis testing (Daily et al., 2007; Nee, 2011, SEM (Rohati et al., 2017; Jabbour, 2015; Chowdhury et al., 2018), MICMAC (Ejdys et al., 2016), TOPSIS (Hessami & Soleimani-Nezhad, 2012), regression analysis (Kaur, 2011; Neves et al., 2017), others descriptive research methods (Babakri et al., 2003; Balzarova et al., 2006; Daily & Huang, 2001; Kitazawa & Sarkis, 2000; Oliveira & Serra Pinheiro, 2009; Price, 2005; Sammalisto & Brorson, 2008; Sujatha & Basu, 2013; Waxin et al., 2020; Zeng et al., 2005). In this study, the application of GTMA enabled consideration of hierarchical relations among those HFs, incorporation of mathematical analytics to quantitively assess the role of each main HFs in the EMS and possession of a sound idea about how much improvement can be brought about. To the authors’ best knowledge, none of these priorly mentioned methodologies could able to perform these tasks. Thus, this study contributes to the existing literature by demonstrating how a GTMA as an operations research tool can be used to consider hierarchical relations among different HFs and assess the roles of those HFs quantitively in an EMS management environment.

Despite being a novel research work establishing the connection between ISO14001 and its crucial HFs for organizations of Bangladesh irrespective of industry nature, this study has some shortcomings. Firstly, the respondents to the questionnaire (that was used to derive the inheritances of the HFs) were from medium to large market size enterprises. Consequently, results may differ while applying the same framework for SMEs from Bangladesh or other emerging economies. Another limitation is not considering the causal relationships among the HFs. Besides, some interdependencies have not been considered resulting in a trade-off with losing a minimal amount of accuracy in the analysis in exchange for increased active involvement of experts.

This study offers some future directions for research. Methodologies can be used to assess the role of HFs in multiple organizations belonging to the same industry to demonstrate, the similarity and contrast of the resulted prioritized HFs and how those similarities and contrasts can be attributed to their relative size, financial capability, geographic dispersion of supply chain and reputation. The concept of this study can also be applied to examine the role of the soft factors solely focusing on SMEs of the emerging economies since these SMEs contribute 60% of total employment and 40% of total GDP there (Ndiaye et al., 2018). Also, researchers can modify the list of HFs and categories of HFs by studying the dynamics of present and future situations or considering differences between the belonging industry and geographical region. They can use different MADM tools, e.g., ANP, interpretive structural modeling, ANP-Decision making trial and evaluation laboratory to evaluate the causal relationships among HR factors. Nevertheless, this research, through its proposed solution methodology and demonstration of the application of this methodology in business enterprises of Bangladesh, attempts to provide generic guidelines about how to measure the role of HFs in any industry from any geographic region at any time. Finally, this study can be replicated to explore the role of HFs in internalizing other certification systems.
